# Extended Phase Unwrapping Max-Flow/Min-Cut Algorithm for Multibaseline SAR Interferograms Using a Two-Stage Programming Approach

**DOI:** 10.3390/s20020375

**Published:** 2020-01-09

**Authors:** Lifan Zhou, Yang Lan, Yu Xia, Shengrong Gong

**Affiliations:** 1 School of Computer Science and Engineering, Changshu Institute of Technology, Changshu 215500, China; cslgxiayu@163.com (Y.X.); shrgong@cslg.edu.cn (S.G.); 2 National Laboratory of Radar Signal Processing, Xidian University, Xi’an 710071, China; lanyangxd@hotmail.com; 3 Collaborative Innovation Center of Information Sensing and Understanding, Xidian University, Xi’an 710071, China

**Keywords:** phase unwrapping (PU), multi-baseline (MB), two-stage programming approach (TSPA), phase unwrapping max-flow/min-cut (PUMA)

## Abstract

Multi-baseline (MB) phase unwrapping (PU) is a key step of MB synthetic aperture radar (SAR) interferometry (InSAR). Compared with the traditional single-baseline (SB) PU, MB PU is applicable to the area where topography varies violently without obeying the phase continuity assumption. A two-stage programming MB PU approach (TSPA) proposed by H. Yu. builds the link between SB and MB PUs, so many existing classical SB PU methods can be transplanted into the MB domain. In this paper, an extended PU max-flow/min-cut (PUMA) algorithm for MB InSAR using the TSPA, referred to as TSPA-PUMA, is proposed, consisting of a two-stage programming procedure. In stage 1, phase gradients are estimated based on Chinese remainder theorem (CRT). In stage 2, a Markov random field (MRF) model of PUMA is designed for modeling local contextual dependence based on the phase gradients obtained by stage 1. Subsequently, the energy of the MRF model is minimized by graph cuts techniques. The experiment results illustrate that the TSPA-PUMA method can drastically enhance the accuracy of the original PUMA method in the rugged area, and is more efficient than the original TSPA method. In addition, the noise robustness of TSPA-PUMA can be improved through adding more interferograms with different baseline lengths.

## 1. Introduction

Interferometric synthetic aperture radar (InSAR) is a powerful tool to reconstruct the digital elevation model (DEM) or surface deformation of the Earth’s surface [1]. Phase unwrapping (PU), as a key processing step of InSAR, is the procedure of retrieving the absolute phase through the wrapped phase. Unfortunately, the traditional single-baseline (SB) PU is an ill-posed problem, i.e., there are infinite solutions to it, if no extra information is added. In fact, a phase continuity assumption (also known as Itoh condition) employed by most SB PU methods is that the absolute value of phase differences between neighboring pixels is less than π [2]. Unfortunately, violent terrain changes and high system noise frequently fail to observe the phase continuity assumption in reality, so it is still difficult for SB PU to generate the correct PU result. However, the multi-baseline (MB) PU problem is well-posed rather than ill-posed, which makes use of the baseline diversity to significantly increase the ambiguity intervals of interferometric phases. To be specific, MB PU can completely eliminate the phase-continuity assumption.

In recent decades, the MB PU has been widely investigated. Yu et al. [3] provided a good review article of MB PU methods, which described that there are mainly two groups of methods: parametric-based and non-parametric-based methods. The main ideas of these two groups of MB PU methods both come from machine-learning technology [3]. The methods in the first group utilize the InSAR probability density function to build a statistical framework based on maximum likelihood (ML) [4,5,6] or maximum a posteriori (MAP) criteria [7,8] to find the MB PU result, [9] provided a good review of the ML- and MAP-based methods, and [10] gave a comparative study of the PU accuracy between the ML- and MAP-based methods. The methods in the second group translate the MB PU problem into an unsupervised learning problem. [11] presented a fast cluster-analysis (CA)-based MB PU method, and [12] further improved it. Besides these two groups of methods, three basic MB PU methods, i.e., the Chinese remainder theorem (CRT)-based method, projection method, and linear combination method, were put forward in [13]. [14] proposed the L∞-norm programming criterion applied to the MB PU. To improve the robustness to noise, [15] presented a closed-form robust CRT method, and [16] put forward a MB PU method based on the mix-integer optimization model. More than that, [17] proposed a Kalman filtering-based MB PU method, and a wavelet approach-based MB PU method was presented in [18]. It should be noted that the major difference between SB and MB PUs lies in their different processing steps. For the detailed implementation of the SB and MB PU methods, the readers can refer to [3].

However, most of the aforementioned MB PU methods suffer from poor noise robustness, and the reason for the noise robustness problem is caused by system noise, surface deformation, or atmospheric effect [3]. In addition, the ML-, MAP-, and CA-based MB PU methods are all based on machine-learning techniques, so they usually need to determine some parameters through some extra information because they do not have clear PU meanings. Under these conditions, these MB PU methods are quite limited in real application. To solve these problems, Yu and Lan [19] proposed a two-stage programming-based MB PU method, abbreviated as TSPA, that formulates a connection between SB and MB PUs, which is also known as TSPA-InSAR technology. In stage 1, TSPA estimates the ambiguity number difference between neighboring pixels using multiple interferograms with different baseline lengths based on the CRT formulation. In stage 2, TSPA obtains the final PU result through using the *L*^1^-norm SB PU method, i.e., minimum-cost flow (MCF) PU method [20]. It is noted that there are several strongly polynomial algorithms that can be applied to solve the MCF model (e.g., minimum mean cycle-canceling algorithm and network simplex algorithm [21]). More than that, some studies indicate that the divide-and-conquer criterion can be used to further reduce the computational and peak memory consumption of the MCF model [22,23]. To further improve the noise robustness of stage 1 of TSPA, [24] proposed a local phase model, which assumes terrain height surface in the neighborhood pixels can be approximated by a plane. Furthermore, [25] used the unscented Kalman filter (UKF) to improve the performance of the stage 2 of TSPA reducing the effect of the noise gradient on the PU results. Furthermore, [26] proposed a technique for applying TSPA to the large-scale MB InSAR data set based on the MB envelope-sparsity theorem. Compared with most of the aforementioned existing MB PU methods, the two main contributions of the TSPA method are listed as follows. First, as a MB PU method, TSPA does not obey the phase continuity assumption by taking advantage of MB diversity. Second, since TSPA makes the link between SB and MB PUs, many existing classical SB PU methods can be transplanted into MB domain.

A SB PU algorithm based on graph cuts, referred to as phase unwrapping max-flow/min-cut (PUMA), was proposed by Bioucas-Dias and Valadao [27]. This algorithm uses a new energy minimization framework, which is based on the Markov random field (MRF). Under this condition, the problem of ambiguity number estimation can be translated into computing a sequence of binary optimizations (i.e., {0, 1}-cut), which can be solved by graph cuts techniques. The reason why this algorithm is so popular is that the MRF model allows a large family of potential functions (i.e., consisting of convex potential and non-convex potential), which gives flexibility to handle effectively both continuous and discontinuous phase features. For convex potentials, the PUMA algorithm exactly solves the classical minimum *L^p^* norm PU problem with *p* ≥ 1. For non-convex potentials, the potentials with exponent less than one with 0 < *p* < 1 have been employed to allow discontinuity preservation [27]. However, as a SB PU algorithm, PUMA is still limited to the phase continuity assumption, so it is potentially hard for the PUMA algorithm to obtain the correct PU result in the discontinuous region. Some researchers have already noticed this issue. [28] extended the PUMA algorithm into MB domain to further increase the discontinuity preserving ability of PUMA, but it is only less influenced by the phase continuity assumption rather than violating the phase continuity assumption. Contrarily, as described earlier, TSPA does not need to satisfy the phase continuity assumption through using two-stage programming. In this case, there is a straightforward idea to transplant the PUMA algorithm into the MB domain using the TSPA approach.

In this paper, an extended PUMA algorithm for MB InSAR using the TSPA approach, abbreviated as the TSPA-PUMA method, is proposed, which consists of a two-stage programming procedure. In stage 1 of TSPA-PUMA, stage 1 of the original TSPA is utilized to estimate the phase gradients based on CRT without obeying the phase continuity assumption. In stage 2 of TSPA-PUMA, an MRF model of PUMA with different types of clique potentials is designed for modeling local contextual dependence based on the phase gradients obtained by stage 1. Subsequently, the energy of MRF model for SB PU is minimized by computing a sequence of binary optimizations solved by graph cuts techniques. This paper uses three simulated InSAR data experiments and two real InSAR data experiments to validate the proposed approach. The results show that the TSPA-PUMA method can significantly improve the PU accuracy of the original PUMA algorithm in the rugged and mountainous area, and the noise robustness of TSPA-PUMA can be improved if employing more interferograms with different baseline lengths.

The rest of this paper is organized as follows. Section 2 reviews the original PUMA method and analyzes its disadvantages of dealing with steep terrain. In Section 3, the TSPA-PUMA method is introduced in detail. Besides that, the noise robustness, time complexity, and parameter selection of TSPA-PUMA are also analyzed. Then, in Section 4, the TSPA-PUMA method is verified by a set of simulated and real MB InSAR datasets and the corresponding experimental results are discussed in detail. Finally, Section 5 concludes this paper.

## 2. Review and Analysis of SB PUMA

### 2.1. Basic Principle of PUMA

In this section, we will review the original PUMA algorithm in SB case. SB PU can be regarded as estimating the unknown integral multiple of 2π to be added at each pixel of the wrapped phase image to restore the absolute phase, given by:

(1) φ(s)=ψ(s)−2k(s)π,

where φ(s) is the wrapped phase of the *s*th pixel, ψ(s) is the unknown absolute phase of the *s*th pixel, and k(s) is the unknown ambiguity number of the *s*th pixel, which is also known as the wrap count. From (1), we can see that directly solving (1) is an ill-posed inverse problem, because there are two unknowns in one equation, i.e., there is no unique solution to (1). Similar to other SB PU methods, the PUMA algorithm also uses the phase continuity assumption to solve this problem. The energy minimization function for PUMA is given by:

(2) $$\begin{array}{cc} \arg & \underset{\begin{array}{c} k\left(s\right) \end{array}}{\min}\;\;\;\underset{\left(s,s-1\right)}{\sum }w\left(s,s-1\right)\cdot V\left({∆}_{\psi }\left(s,s-1\right)\right),\;\; \end{array}$$

where the indexes s and s−1 denote two neighboring pixels and w(s,s−1) is the weighted coefficient, which can be derived from any kind of quality map in InSAR [29]. V(·) is clique potential, defined by $V\left(\cdot \right)={\left(\cdot \right)}^{p}$, and p is the potential exponent, which determines how the phase of the neighboring pixels in the clique interact. Note that changes of the MRF model of PUMA depend primarily on choosing different clique potential V(·). When the corresponding clique potentials are convex (i.e., p≥1), PUMA exactly solves the classical $L^{p}$ minimum norm PU problem. In the case p=2, PUMA will become the least square method. A drawback of the $L^{2}$-norm clique potential is that it tends to smooth discontinuities. $L^{1}$-norm clique potential (p=1) performs better than $L^{2}$-norm clique potential in preserving discontinuities. The major advantage of PUMA lies its non-convex clique potential with 0<p<1, which allows an increased probability of sharp transitions. ${∆}_{\psi }\left(s,s-1\right)$ is the absolute phase gradients, i.e., the absolute phase difference of the neighboring pixels, which is defined by:

(3) $${∆}_{\psi }\left(s,s-1\right)=\Delta_{\varphi }\left(s,s-1\right)+2\pi \cdot \left(k\left(s\right)-k\left(s-1\right)\right),$$

where ${∆}_{\varphi }\left(s,s-1\right)$ is the wrapped phase differences of the neighboring pixels. The PUMA algorithm aims to estimate the wrap count  k(s) that minimizes the phase gradients ${∆}_{\psi }\left(s,s-1\right)$ obtained by Equation (2), which can be regarded as a binary optimization problem. Initially, the labels of all pixels are set to zero, i.e., $k^{t=0}\left(s\right)=0$. At each iteration step, every pixel’s label would either be 1 or 0, i.e., $k^{t+1}\left(s\right)=k^{t}\left(s\right)+\delta^{t+1}\left(s\right)$, in which the *t* denotes iteration and $\delta^{t+1}\left(s\right)\in \left\{0,1\right\}$, meaning that every pixel’s label either increases by 1 (phase plus 2π) or 0 (phase remains unchanged). Every iteration aims to decrease the value of the energy function of Equation (2) as much as possible. After each iteration, the unwrapped phase is updated, i.e., $\;\psi^{t+1}\left(s\right)=\varphi \left(s\right)+2\pi \cdot k^{t+1}\left(s\right)$, and the energy function of Equation (2) is recalculated. When the energy ceases to decrease, the iteration is terminated, where the unwrapped phase is estimated, i.e., $\psi^{t=\mathrm{end}}\left(s\right)=\varphi \left(s\right)+2\pi \cdot k^{t=\mathrm{end}}\left(s\right)$. The binary optimization problem in the above referred sequence can be solved by graph cuts from [30], which are computed efficiently using max-flow/min-cut algorithms. For the convex clique potential (p≥1), because it satisfies the regularity condition, this binary optimization problem can be solved exactly using the standard graph cuts algorithm. With respect to the non-convex clique potential (0<p<1), because it does not obey the regularity condition, it is impossible to minimize the energy function of Equation (2) via the standard graph cuts algorithm. To solve this issue, an approximate version of the graph cuts algorithm is devised by applying majorize-minimize (MM) approximation, which can cope with the local minima arising from non-convex potentials. For the detailed implementation of graph cuts-based optimization of the energy function of Equation (2), the readers can refer to [27].

### 2.2. Problem Analysis

**Figure 1:**
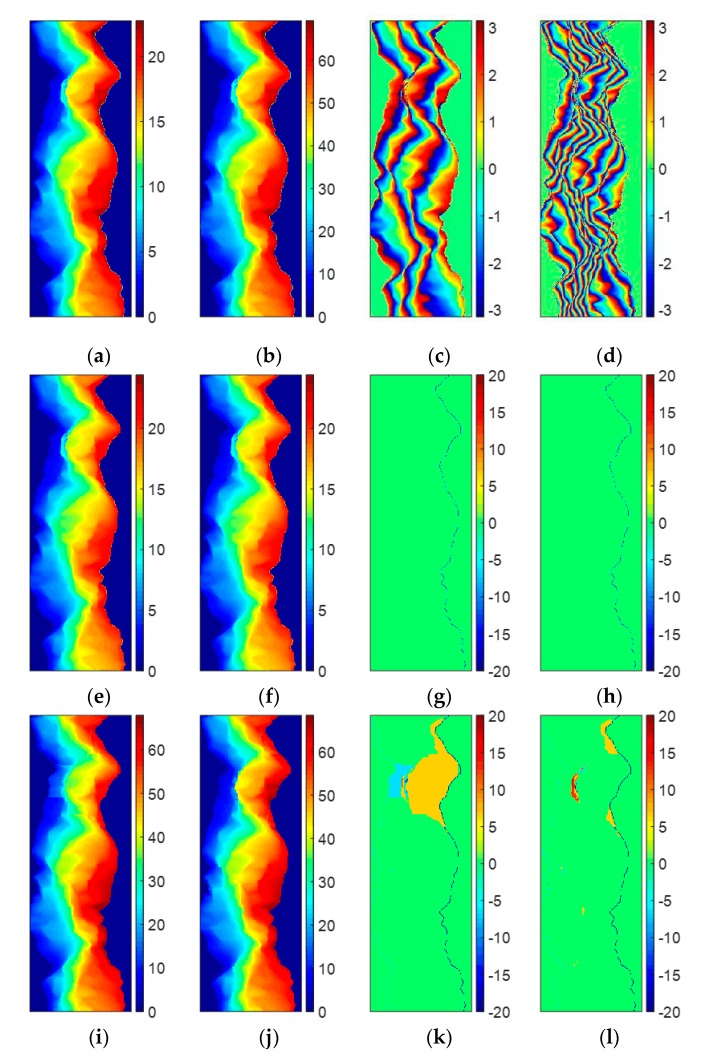
(**a**,**b**) Reference unwrapped phases ((**a**) short and (**b**) long baseline length). (**c**,**d**) Simulated wrapped phases of (**a**,**b**). (**e**,**f**) PU results of (**c**) obtained by (**e**) PUMA (clique potential exponent is 1), and (**f**) PUMA (clique potential exponent is 0.5). (**g**,**h**) Errors between (**a**) and PU results (**e**,**f**). (**i**,**j**) PU results of (**d**) obtained by (**i**) PUMA (clique potential exponent is 1), and (**j**) PUMA (clique potential exponent is 0.5). (**k**,**l**) Errors between (**b**) and PU results (**i**,**j**).

As described above, the PUMA algorithm aims to estimate the wrap count k(s) that minimizes the phase gradients ${∆}_{\psi }\left(s,s-1\right)$ obtained by Equation (2) according to the phase continuity assumption. From Equation (2), we can see that the credibility of the PU result of PUMA is directly related to the correctness of ${∆}_{\psi }\left(s,s-1\right)$. Unfortunately, violent topographic changes and high system noise frequently make the phase continuity assumption does not work well. Under this condition, it is difficult to obtain the correct ${∆}_{\psi }\left(s,s-1\right)$ from the phase continuity assumption. Therefore, if the accuracy of ${∆}_{\psi }\left(s,s-1\right)$ is too low, no matter what kind of clique potential V(·) is employed, it could be impossible for the PUMA algorithm to obtain the full correct PU solution. For example, Figure 1a,b show the reference unwrapped phases with two different baselines, which come from the mountainous area around the Isolation Peak region of Colorado [31]. Figure 1c,d show two simulated noise-free interferograms of Figure 1a,b. Table 1 illustrate the major parameters of the simulated system. Figure 1e,f show the PU results of Figure 1c obtained by the PUMA methods with clique potential exponent 1 and 0.5, respectively. Figure 1g,h are the errors between Figure 1a and Figure 1e,f, respectively. Figure 1i,j show the PU results of Figure 1d obtained by the PUMA methods with clique potential exponent 1 and 0.5, and the corresponding errors between Figure 1b and Figure 1i,j are shown in Figure 1g,h, respectively. To fairly evaluate the PU results, the same reference point and range of the color bar are used in the PU results obtained by the two PUMA methods of the same interferogram, respectively (similarly hereinafter in experiments 1, 2 3, 4, and 5). Because the pattern of the fringes in Figure 1c is simple, we can see that the two PUMA methods with clique potential exponent 1 and 0.5 both obtain the correct PU results. However, when the pattern of the fringes in Figure 1d becomes very complicated which is difficult for the PU process, the PU accuracy of these two methods will significantly decrease. The reason is that the pattern of the fringes in Figure 1d changes fiercely, which makes the failure of the phase continuity assumption, i.e., the absolute phase differences between neighboring pixels are larger than π. Under this condition, even if PUMA with non-convex potential is better than that with convex potential due to its discontinuity preserving ability, it is still difficult enough for PUMA with non-convex potential to perform correctly. Therefore, it can be seen that the PUMA method can find the correct PU result in the area where topography is comparative flat, but in the area where topography jumps more drastically, PUMA cannot find the correct PU solution anymore, no matter what kind of clique potential is chosen.

## 3. TSPA-PUMA Methodfor MB PU

**Figure 2:**
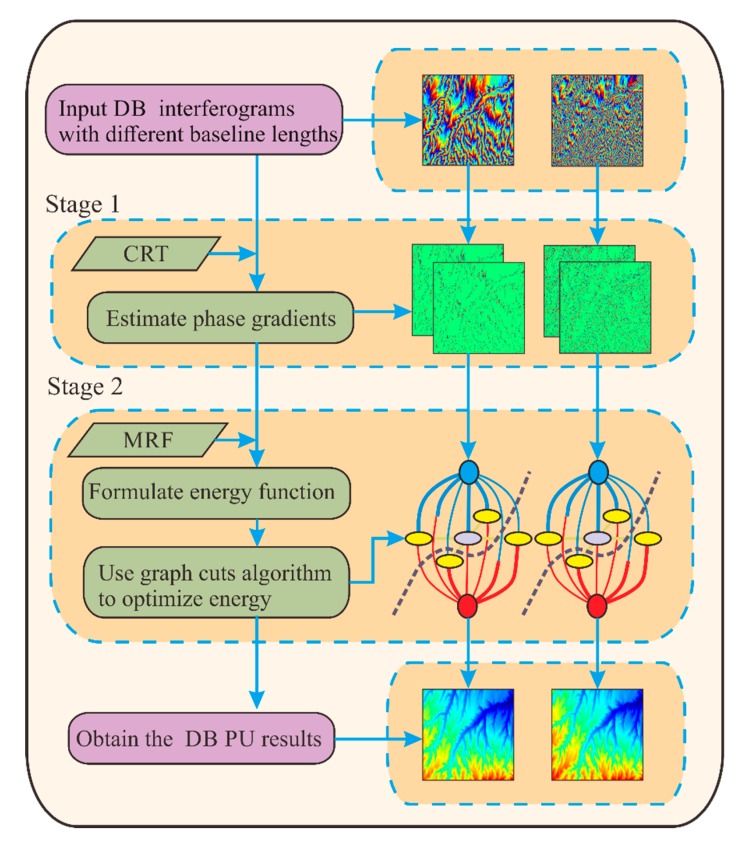
Schematic representation of the proposed TSPA-PUMA method.

According to the discussion in Section 2, we conclude that the traditional PUMA algorithm is limited to the phase continuity assumption. In this Section, we will introduce the proposed TSPA-PUMA method which can break through the limitation of the phase continuity assumption. In this Section, we only consider the dual-baseline (DB) case for simplicity, and the MB case can be extended easily. A schematic representation of the proposed TSPA-PUMA is illustrated in Figure 2. In the following, we will introduce the two stages in the TSPA-PUMA method in detail.

### 3.1. Stage 1: Estimating the Phase Gradient

The DB InSAR measurement of a pixel case can be given by:

(4) $$\varphi_{r}\left(s\right)=\psi_{r}\left(s\right)-2k_{r}\left(s\right)\cdot \pi ,$$

where $\varphi_{r}\left(s\right)$, $\psi_{r}\left(s\right)$ and $k_{r}\left(s\right)$ are the wrapped phase, absolute phase, and ambiguity number of the *s*th pixel in interferogram r (r=1,2), respectively. $\varphi_{r}\left(s\right)$ can be measured by the DB InSAR system, but $\psi_{r}\left(s\right)$ and $k_{r}\left(s\right)$ are the unknowns in one equation that need to be solved. If the ambiguity number $k_{r}\left(s\right)$ of each pixel in two interferograms can be solved, $\psi_{r}\left(s\right)$ can be obtained through Equation (4). The absolute phases of the two interferograms can be calculated by using the baseline lengths such as [19]:

(5) $$B_{2}\cdot \left(\varphi_{1}\left(s\right)+2\pi \cdot k_{1}\left(s\right)\right)=B_{1}\cdot \left(\varphi_{2}\left(s\right)+2\pi \cdot k_{2}\left(s\right)\right),$$

where $B_{1}$ and $B_{2}$ represent two different normal baseline (also known as perpendicular baseline) lengths. In this paper, normal baseline length is abbreviated as baseline length. According to Equation (5), the TSPA-PUMA method maintains the stage 1 of the TSPA, which builds the relationship of phase gradient information in different interferograms with different baseline lengths, given by:

(6) $$B_{2}\cdot \left({∆}_{\varphi_{1}}\left(s,s-1\right)+2\pi \cdot {\hat{∆}}_{k_{1}}\left(s,s-1\right)\right)=B_{1}\cdot \left({∆}_{\varphi_{2}}\left(s,s-1\right)+2\pi \cdot {\hat{∆}}_{k_{2}}\left(s,s-1\right)\right),$$

where ${∆}_{\varphi_{1}}\left(s,s-1\right)\;$and ${∆}_{\varphi_{2}}\left(s,s-1\right)$ are the wrapped phase differences between neighboring pixels of interferogram *r* (*r* = 1, 2), ${\hat{\Delta }}_{k_{1}}\left(s,s-1\right)$ and ${\hat{\Delta }}_{k_{2}}\left(s,s-1\right)$ are the ambiguity number gradient between neighboring pixels of interferogram *r*. Note that there are two directions (vertical and horizontal) of neighboring pixels for ${\hat{\Delta }}_{k_{r}}\left(s,s-1\right)$ and $\Delta_{\varphi_{r}}\left(s,s-1\right)$. Because ${\hat{\Delta }}_{k_{1}}\left(s,s-1\right)$ and ${\hat{\Delta }}_{k_{2}}\left(s,s-1\right)$ belong to the integer, we can obtain the solution to Equation (6) under some special combination of the baseline lengths according to CRT [19]. Equation (6) can be solved by the optimization model given by:

(7) $$\begin{array}{cc} \arg & \underset{\begin{array}{c} {\hat{\Delta }}_{k_{r}}\left(s,s-1\right) \end{array}}{\min}\;\;\;\;\;\;\;\;\;\left|h\left(s,s-1\right)\right|\;\;\;\;\;\;\;\;\;\;\;\;\;\;\;\;\;\;\\ s.t. & {\hat{\Delta }}_{k_{r}}\left(s,s-1\right)\in \mathrm{integer},r=1,2, \end{array}$$

where ${\hat{\Delta }}_{k_{r}}\left(s,s-1\right)\;$are the decision variables of interferogram *r*. It is noted that ${\hat{\Delta }}_{k_{r}}\left(s,s-1\right)$ can be larger than 1 or less than −1, which implies that the phase continuity assumption does not need to be satisfied (the phase continuity assumption only allows ${\hat{\Delta }}_{k_{r}}\left(s,s-1\right)$ to be ±1 or 0). h(s,s−1) is the auxiliary variables, defined by:

(8) $$h\left(s,s-1\right)=B_{2}\cdot \left(\Delta_{\varphi_{1}}\left(s,s-1\right)+2\pi \cdot k_{1}\left(s\right)\right)-B_{1}\cdot \left(\Delta_{\varphi_{2}}\left(s,s-1\right)+2\pi \cdot k_{2}\left(s\right)\right).$$

It can be seen that h(s,s−1) is the CRT bias, so Equation (8) is to find the ambiguity number gradient$\;{\hat{\Delta }}_{k_{r}}\left(s,s-1\right)$ with minimum CRT bias [19]. Under this condition, the phase gradient ${\hat{\Delta }}_{\psi_{r}}\left(s,s-1\right)$ of interferogram *r* can be estimated by:

(9) $${\hat{\Delta }}_{\psi_{r}}\left(s,s-1\right)=\Delta_{\varphi_{r}}\left(s,s-1\right)+2\pi \cdot {\hat{\Delta }}_{k_{r}}\left(s,s-1\right).$$

### 3.2. Stage 2: Unwrapping the Phase Gradient Using Graph Cuts Algorithm

Based on the gradient information obtained by Equation (9), the energy minimization framework based on the MRF model for TSPA-PUMA respectively obtain the final PU solution of each interferogram *r*, which is obtained by Equation (10),

(10) $$\begin{array}{cc} \arg & \underset{\begin{array}{c} k_{r}\left(s\right) \end{array}}{\min}\;\;\;\;\;\;\sum_{\left(s,s-1\right)}w_{r}\left(s,s-1\right)\cdot V\left(\Delta_{\psi }{}_{r}\left(s,s-1\right)-{\hat{\Delta }}_{\psi_{r}}\left(s,s-1\right)\right),\;\;\;\;\;\;\;\;\;\;\;\;\;\;\;\;\;\;\; \end{array}$$

where $w_{r}\left(s,s-1\right)$ is the weighted coefficient of interferogram *r*, and $k_{r}\left(s\right)$ is the decision variable of interferogram *r*. From Equation (10), it can be seen that the aim of TSPA-PUMA is to minimize the difference between the absolute phase gradients $\Delta_{\psi r}\left(s,s-1\right)$ and the estimated gradients ${\hat{\Delta }}_{\psi_{r}}\left(s,s-1\right)$ obtained from stage 1 of TSPA-PUMA. Compared with the traditional PUMA algorithm which obeys the phase continuity assumption, the major improvement of TSPA-PUMA is that it does not need to follow the assumption, because the ambiguity number gradient ${\hat{\Delta }}_{k_{r}}\left(s,s-1\right)$ obtained by Equation (7) can be larger than 1 or less than −1. If we transform the phase gradients $\Delta_{\psi }\left(s,s-1\right)$ obtained by the Equation (3) into DB case, we will obtain:

(11) $$\Delta_{\psi }{}_{r}\left(s,s-1\right)=\Delta_{\varphi r}\left(s,s-1\right)+2\pi \cdot \left(k_{r}\left(s\right)-k_{r}\left(s-1\right)\right).$$

Then, if we substitute Equations (9) and (11) into Equation (10), the energy minimization framework for TSPA-PUMA can be rewritten to:

(12) $$\begin{array}{cc} \text{arg} & \underset{\begin{array}{c} k_{r}\left(s\right) \end{array}}{\min}\;\;\;\;\sum_{\left(s,s-1\right)}w_{r}\left(s,s-1\right)\cdot V\left(k_{r}\left(s\right)-k_{r}\left(s-1\right)-{\hat{\Delta }}_{k_{r}}\left(s,s-1\right)\right),\;\; \end{array}$$

where $k_{r}\left(s\right)$ (r=1,2) are solutions to Equation (12) of the two different interferograms r. Because optimization of $k_{1}\left(s\right)$ and $k_{2}\left(s\right)$ is independent of each other, we can optimize them separately. Similar to the PUMA algorithm, the minimization of the energy function of TSPA-PUMA obtained by Equation (12) can be regarded as a jump-move optimization problem. It is worth mentioning that, with respect to TSPA, the innovative part of TSPA-PUMA lies in stage 2, where the graph cuts algorithm is used to optimize the energy function of (12). Initially, the ambiguity number of the *s*th pixel in interferogram r is set to zero, i.e., $k_{r}^{t=0}\left(s\right)$ = 0. At each iteration, every ambiguity number of the *s*th pixel in interferogram r either increases by one (i.e., the ambiguity number pluses one) or zero (i.e., the ambiguity number remains unchanged), that is, $k_{r}^{t+1}\left(s\right)$ = $k_{r}^{t}\left(s\right)+\delta_{r}^{t+1}\left(s\right)$, where $\delta_{r}^{t+1}\left(s\right)\in \left\{0,1\right\}$. For each pair of neighboring pixels (s,s−1) in interferogram r, the clique potential to be minimized is defined as:

(13) $$E\left(\delta_{r}^{t+1}\left(s\right),\delta_{r}^{t+1}\left(s-1\right)\right)=V\left(k_{r}^{t}\left(s\right)-k_{r}^{t}\left(s-1\right)-{\hat{\Delta }}_{k_{r}}\left(s,s-1\right)+\delta_{r}^{t+1}\left(s\right)-\delta_{r}^{t+1}\left(s-1\right)\right).$$

For the convex clique potential (p≥1), the clique potential obtained by (13) satisfies the regularity condition, so the standard graph cuts algorithm can be used to optimize them. For the non-convex clique potential (0<p<1), the MM concept [27] is employed to make the non-convex clique potential obtained by (14) obey the regularity condition, so they can also be optimized by the standard graph cuts algorithm. According to (13), we have:

(14) $$\begin{array}{c} E\left(0,0\right)=V\left(k_{r}^{t}\left(s\right)-k_{r}^{t}\left(s-1\right)-{\hat{\Delta }}_{k_{r}}\left(s,s-1\right)\right)\\ E\left(1,1\right)=V\left(k_{r}^{t}\left(s\right)-k_{r}^{t}\left(s-1\right)-{\hat{\Delta }}_{k_{r}}\left(s,s-1\right)\right)\\ E\left(0,1\right)=V\left(k_{r}^{t}\left(s\right)-k_{r}^{t}\left(s-1\right)-{\hat{\Delta }}_{k_{r}}\left(s,s-1\right)-1\right)\\ E\left(1,0\right)=V\left(k_{r}^{t}\left(s\right)-k_{r}^{t}\left(s-1\right)-{\hat{\Delta }}_{k_{r}}\left(s,s-1\right)+1\right). \end{array}$$

Considering all pairs of neighboring pixels, the energy minimization function of each binary iteration is given by:

(15) $$\begin{array}{cc} \arg & \underset{\begin{array}{c} \delta_{r}^{t+1}\left(s\right) \end{array}}{\min}\;\;\;\;\sum_{\left(s,s-1\right)}w_{r}\left(s,s-1\right)\cdot E\left(\delta_{r}^{t+1}\left(s\right),\delta_{r}^{t+1}\left(s-1\right)\right).\;\;\;\; \end{array}$$

**Figure 3:**
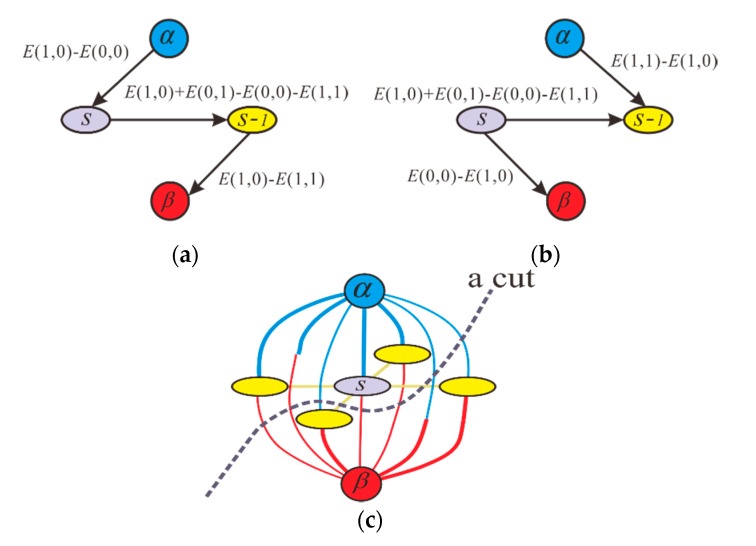
The elementary graph is constructed, where α and β represent two terminals and (s,s−1) represent the two neighboring pixels. (**a**) In the case of E(1,0)−E(0,0)>0 and E(1,1)−E(1,0)>0. (**b**) In the case of E(1,0)−E(0,0)<0 and E(1,1)−E(1,0)<0. (**c**) A main graph is obtained by merging the two elementary graphs, where an α−β cut is a set of edges such that the terminals are separated into two disjoint sets α∈1 (the ambiguity number pluses one) and β∈0 (the ambiguity number remains unchanged).

The minimization of (15) can be achieved through a cut on the weighted graph *σ* =〈υ,ϵ〉 with two terminals α and β. The set of vertices υ represent the pixels in each interferogram, and the set of edges ϵ denote the pairs of neighboring vertices in each interferogram. An α−β cut is a set of edges such that the terminals are separated into two disjoint sets α∈1, i.e., the ambiguity number pluses one, and β∈0, i.e., the ambiguity number remains unchanged. The cost of the cut equals the sum of its clique potential between α and β. Then, we construct the elementary graph for each clique potential, as shown in Figure 3a,b. From Figure 3a,b, it can be seen that the directed edge (s,s−1) is assigned a weight of E(0,1)+E(1,0)−E(0,0)−E(1,1). Moreover, for vertex s, if E(1,0)−E(0,0)>0, then the edge (α,s) is assigned a weight of E(1,0)−E(0,0); otherwise, the edge (s,β) is assigned a weight of E(0,0)−E(1,0). Similarly, for the neighboring vertex s−1, if E(1,1)−E(1,0)>0, then the edge (α,s−1) is assigned a weight of E(1,1)−E(1,0); otherwise, the edge (s−1,β) is assigned a weight of E(1,0)−E(1,1). Finally, the two elementary graphs are merged to obtain a main graph, as shown in Figure 3c. At every jump-move iteration, the minimum cut problem attempts to find the cheapest cut among all cuts separating the terminals, which can be obtained using the max-flow algorithm. That is to say, every jump-move iteration is intended to reduce the value of the energy function of (15) as much as possible. When the energy ceases to decrease, the binary jump move is terminated. Finally, we can obtain the DB PU results, i.e., $\psi_{r}^{t=end}\left(s\right)$ (r=1,2), which is equal to $\varphi_{r}\left(s\right)+2\pi \cdot k_{r}^{t=end}\left(s\right)$ (r=1,2).

### 3.3. Analysis of the Noise Robustness

It should be noted that stage 1 of TSPA-PUMA is dependent on CRT, which is too sensitive to measurement bias that is potentially caused by some decorrelation factors, e.g., atmospheric effect or co-registration error, etc. Considering the atmospheric artifact, this usually shows a strong spatial correlation [32]. Hence, the effect of atmosphere on the wrapped phases of neighboring pixels should be close to each other. Because Equation (6) uses the information of wrapped phase difference between neighboring pixels, the effect of atmosphere could be counteracted in Equation (6). Therefore, stage 1 of the TSPA-PUMA method does not fear the atmospheric effect. However, it is still sensitive to the noise levels caused by other decorrelation components. Under this condition, the incorrect phase gradient information obtained in stage 1 will reduce the accuracy of final PU result directly. Unlike [24,25] both using filtering-based methods to alleviate the effects of the phase noise on the estimated phase gradients, in this paper, we resist the influence of the noise in stage 1 of TSPA-PUMA through using the MB InSAR dataset with different baseline lengths. To be specific, the more interferograms are involved to estimate the phase gradients based on the CRT formulation, the higher accuracy on ambiguity number gradient estimation will be obtained (it is because that more observed samples of interferometric phases from different interferograms with different baseline lengths are involved, more phase noise can be ignored). Therefore, TSPA-PUMA has good noise robustness if we utilize enough interferograms. In Section 4.2, we will validate the noise robustness of TSPA-PUMA using the MB InSAR system with different baseline lengths.

### 3.4. Analysis of the Time Complexity

It should be noted that the main running time and memory consumption of TSPA-PUMA lies in stage 2, which is similar to TSPA. In addition, the computational complexity of stage 2 of the TSPA-PUMA method is close to that of the original PUMA method, due to their similar optimization strategy. The time complexity of TSPA-PUMA is R·K·T(n,m), where R is the number of the interferogram (i.e., R=2 in DB case), K is the number 2π of multiples (i.e., the number of iterations) and T(n,m) is the complexity of a max-flow computation in a graph with n nodes and m edges in one interferogram. Regarding memory usage, TSPA-PUMA requires R·7n bytes. We observe that the computational burden of TSPA-PUMA lies in computing the max-flow algorithm. However, the max-flow solution in the graph cuts algorithm has potential for parallelization, which is suitable for GPU acceleration [33]. Under this condition, the time efficiency of TSPA-PUMA can be increased drastically. Therefore, it can be seen that the total time and space complexities of TSPA-PUMA are practical.

### 3.5. Analysis of the Parameter Selection

Note that TSPA-PUMA requires only one parameter, i.e., the potential exponent p in stage 2, to be chosen. The potential exponent  p in TSPA-PUMA is similar to that in the traditional PUMA method, which defines how the phase of the neighboring pixels in the clique interact [27]. As mentioned earlier, if p≥1, i.e., using the convex potential, PUMA can find the correct PU result in the flat area. If 0<p<1, i.e., using the non-convex potential, PUMA has phase discontinuity preserving ability in the rugged area. However, in the TSPA-PUMA method, the meaning of potential exponent  p seems to be completely different. The reason is that the phase gradients estimated by stage 1 of TSPA-PUMA can violate the phase continuity assumption, so stage 2 of TSPA-PUMA does not need to use non-convex potential to preserve the phase discontinuity. On the contrary, the smaller the potential exponent p is, the lower accuracy on the final PU result will be obtained (it is because that nonconvex potential grows much slower than the convex potential, so it allows strong phase noise not to be penalized too much). Similarly, the larger  p the potential exponent is, the accuracy of the final PU result will also be reduced. This is because, when p>1, TSPA-PUMA allows the high-quality regions to share the phase gradient error from the noisy region. According to experimental results, we observe that p=1, i.e., $L^{1}$-norm model, is the best parameter for the TSPA-PUMA method not only in the discontinuous area but also in the noisy region. In Section 4.5, some detailed experiments on the effect of the potential exponent  p will be presented.

## 4. Performance Analysis

In this Section, the TSPA-PUMA method is compared with the original PUMA and TSPA methods through five independent experiments from different aspects. The source codes of PUMA and TSPA are both from their algorithm designers [34,35]. The implementation environment of these three methods is MATLAB. Note that the clique potential exponent p of TSPA-PUMA is set to 1 (to be kept in experiments 1–4), and the reason will be given in Section 4.5. The first experiment tests the PU performance of the TSPA-PUMA method using the simulated DB InSAR dataset in the terrain with the abrupt change. The second experiment examines the noise robustness of TSPA-PUMA when applied to a simulated MB InSAR dataset with eight interferograms. The third one verifies TSPA-PUMA through using a real TanDEM-X DB InSAR dataset with two interferograms. The fourth one examines the effectiveness of TSPA-PUMA in a real InSAR MB dataset of ALOS PALSAR with four interferograms. The last one explores the effect of the potential exponent p on the TSPA-PUMA method.

### 4.1. Experiment 1

**Figure 4:**
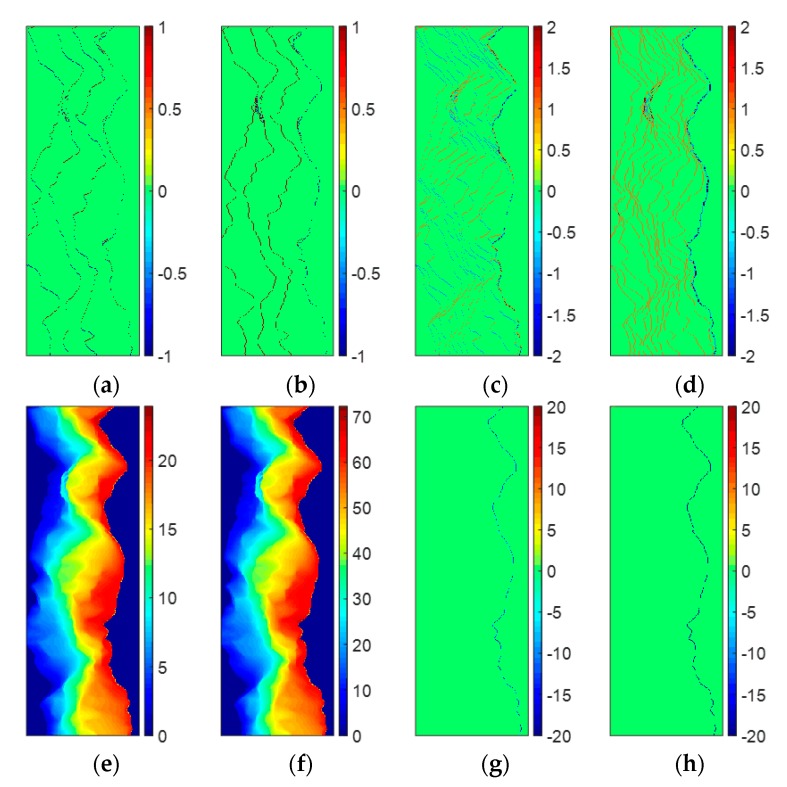
(**a**,**b**) Vertical and horizontal neighboring ambiguity number differences of Figure 1c. (**c**,**d**) Vertical and horizontal neighboring ambiguity number differences of Figure 1d. (**e**) PU results of Figure 1c obtained by TSPA-PUMA. (**f**) PU results of Figure 1d obtained by TSPA-PUMA. (**g**) Errors between Figure 1a and PU results (**e**). (**h**) Errors between Figure 1b and PU results (**f**).

The first experiment is also performed on the simulated DB InSAR dataset which is shown in Figure 1c,d. Figure 4a,b illustrate the vertical and horizontal ambiguity number differences between neighboring pixels of Figure 1c, estimated by stage 1 of TSPA-PUMA, respectively. Figure 4c,d are the vertical and horizontal ambiguity number differences between neighboring pixels of Figure 1d, estimated by stage 1 of TSPA-PUMA, respectively. From Figure 4a,b, we can observe that the estimated ambiguity number differences are restricted to ±1 and 0, because the fringe of Figure 1c does not fiercely change, so the phase continuity assumption still holds well. From Figure 4c,d, it can be observed that some ambiguity number differences are larger than 1 or less than −1, meaning that the phase continuity assumption does not hold any more, due to the fringe of Figure 1d with the violent change. Based on these phase gradients, TSPA-PUMA can overcome the limitation of the phase continuity assumption. Figure 4e shows the PU result of Figure 1c obtained by TSPA-PUMA by using the gradient information shown in Figure 4a,b, and Figure 4g shows the errors between Figure 1a and Figure 4e. Figure 4f is the PU result of Figure 1d obtained by TSPA-PUMA by using the gradient information shown in Figure 4c,d, and Figure 4h shows the errors between Figure 1b and Figure 4f. From Figure 4g, we observe that TSPA-PUMA can generate the correct PU result on short baseline as same as the PUMA method. From Figure 4h, it can be noticed that TSPA-PUMA gives us a flawless PU result using the phase gradient information of Figure 4c,d. The statistical information of Figure 1k,j, Figure 4g,h is shown in Table 2, where the root mean-square error (abbreviated as RMSE) of the PU accuracy is given by:

(16) $$\begin{array}{c} \eta \end{array}=\sqrt{\frac{1}{L}\cdot {∥\hat{\Psi }-\Psi ∥}^{2}}$$

where Ψ is the vector collecting from the reference unwrapped phase, $\hat{\Psi }$ is the vector collecting from the estimated unwrapped phase, L is the length of the vector Ψ and $\hat{\Psi }$, and ${∥\cdot ∥}^{2}$ is the quadratic norm. It is worth mentioning that the units of Ψ and $\hat{\Psi }$ are both radian in this paper. From Table 2, it can be seen that RMSEs of Figure 1g,h and Figure 4g are about 0.9, meaning that the three methods all obtain the correct PU solution of an interferogram with short baseline length. In addition, for the interferogram with long baseline length, we observe that TSPA-PUMA generates the lower RMSE of Figure 4h than those of Figure 1k,l obtained by PUMA with potential exponent 0.5 and 1. Therefore, we can conclude that the TSPA-PUMA method is more effective in the terrain with abrupt change than the original PUMA method.

### 4.2. Experiment 2

**Figure 5:**
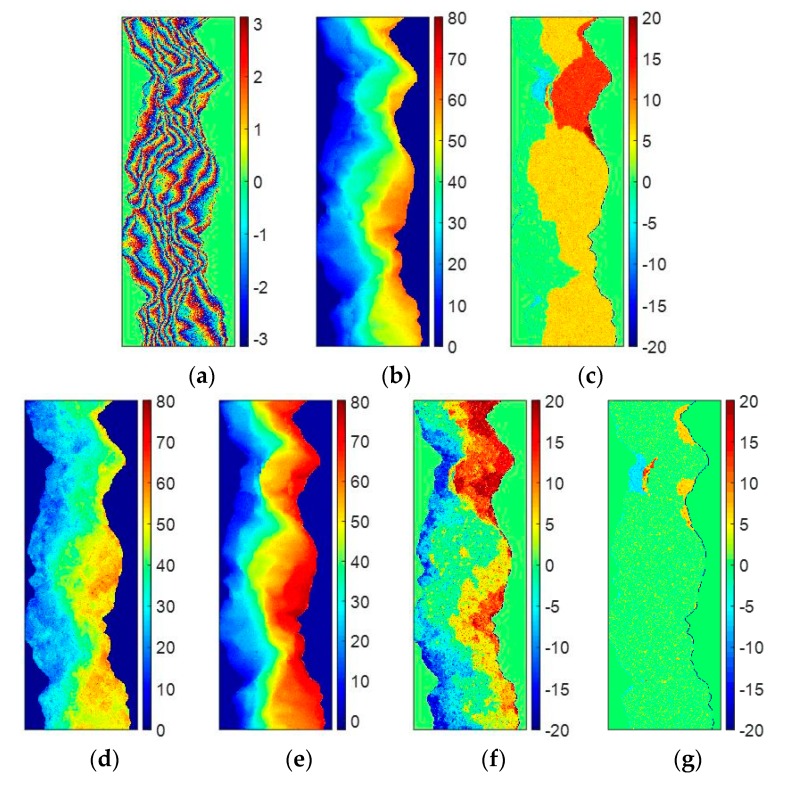
(**a**) Simulated wrapped phases of Figure 1b (coherence coefficient is 0.75). (**b**) PU results of (**a**) obtained by PUMA (clique potential exponent is 0.5). (**c**) Errors between Figure 1b and PU results (**b**). (**d**,**e**) PU results of (**a**) obtained by (**d**) DB TSPA-PUMA and (**e**) MB TSPA-PUMA. (**f**,**g**) Errors between Figure 1b and PU results (**d**,**e**).

The second experiment is also conducted on the simulated interferogram (baseline length is 330 m) which is shown in Figure 1d. To examine the noise robustness of TSPA-PUMA, some phase noise is added according to the employed probability density function of the noise wrapped phase in [36]. It is worth mentioning that, in our simulation, we use the general coherence coefficient to jointly express all the decorrelation components, e.g., atmosphere effect or co-registration error, etc. Figure 5a shows the simulated interferogram, and the mean coherence coefficient of Figure 5a is 0.75. The reference unwrapped phase of Figure 5a is Figure 1b. From Figure 5a, it can be found that, due to the low coherence, the phase fringes are very complicated and PU becomes very tough. Figure 5b is the PU result of the original PUMA method with potential exponent 0.5, and the corresponding errors between Figure 5b and Figure 1b are shown in Figure 5c. We can see that several discontinuous variations are seen clearly in Figure 5c, and RMSE of Figure 5c is up to 9.5992. The reason is that low coherence of interferogram of Figure 5a aggravates the fringe blurrier, which destroy the phase continuity assumption, so it is hard for the SB PU methods to perform correctly. Figure 5d is the PU result of the TSPA-PUMA method based on the DB InSAR dataset used in experiment 1, whose parameters are listed in Table 1. Figure 5f is the corresponding errors between Figure 5d and Figure 1b. From Figure 5f, we find that TSPA-PUMA using the DB InSAR dataset also has obvious unwrapping errors in the phase image, and the RMSE of Figure 5f is 7.6592. The reason is that, although TSPA-PUMA does not obey the phase continuity assumption in the rugged area, stage 1 of TSPA-PUMA is sensitive to noise level which produces the incorrect phase gradient information. In this case, TSPA-PUMA cannot obtain the correct PU result where the fringe is polluted by high noise. Unlike [24,25] who apply the filter-based methods to suppress the influence of incorrect phase gradients obtained by stage 1 of TSPA-PUMA, in this paper, we utilize MB InSAR dataset with more interferograms to remove the phase gradient errors. Major parameters of the MB InSAR system are the same as that used in experiment 1 which is listed in Table 1, but this time, eight interferograms with different baseline lengths are used to perform the TSPA-PUMA method (baseline lengths are 70 m, 150 m, 330 m, 471 m, 550 m, 631 m, 753 m and 831 m, respectively). It should be noted that the number of baselines used in TSPA-PUMA could be any value theoretically, if the ratio of baseline lengths of different interferograms satisfies the CRT formulation. However, CRT is sensitive to the baseline length. In other words, different baseline lengths could result in different performances of TSPA-PUMA. A baseline design criterion was proposed by [37] to determine the optimal baseline length for MB PU. In this experiment, the choice of eight baseline lengths satisfies the baseline design criterion proposed in [37]. Figure 5e is the PU result generated by TSPA-PUMA using MB InSAR dataset, and the corresponding errors between Figure 5e and Figure 1b is illustrated in Figure 5g. From Figure 5g, we observe that the TSPA-PUMA method using the MB InSAR dataset alleviates most of unwrapping errors in the PU result, and RMSE of Figure 5g is 3.4297, which is much lower than the former two methods. This is because that more interferograms are involved in stage 1 of TSPA-PUMA, the higher accuracy on ambiguity number gradient estimation will be obtained. Under this condition, the noise robustness of TSPA-PUMA can be improved drastically.

To further research into the relationship between the number of interferograms and the noise robustness of TSPA-PUMA, we utilize seven MB InSAR datasets with different number of interferograms between 2 and 8 with an increment of 1 to perform the TSPA-PUMA method. The relationship between the estimation RMSE of TSPA-PUMA and the number of interferograms is tabulated in Table 3. From Table 3, it can be observed clearly that the RMSE of the TSPA-PUMA performance can be decreased with the number of interferograms increasing. That is to say, the noise robustness of TSPA-PUMA can be enhanced through using more interferograms, because when more observed samples of interferometric phases are involved, the phase noise can be reduced. Therefore, we can see that TSPA-PUMA has good noise robustness if we utilize enough interferograms with different baseline lengths.

### 4.3. Experiment 3

**Figure 6:**
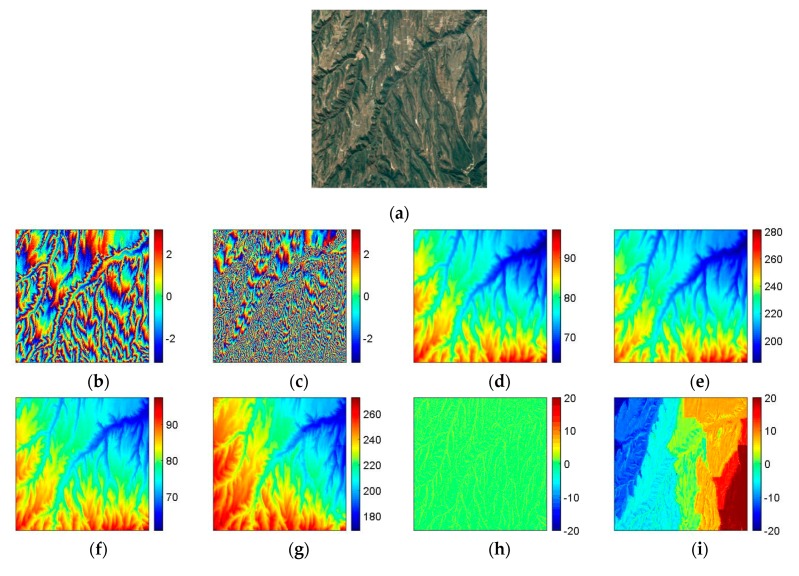

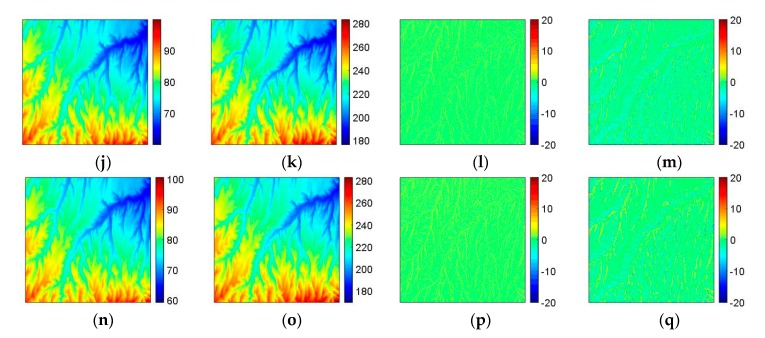
(**a**) Google Earth image of the study area. (**b**,**c**) TanDEM-X interferograms with different baseline lengths ((**b**) short and (**c**) long baseline length). (**d**,**e**) The reference unwrapped phases of (**b**,**c**). (**f**,**g**) PU results of (**b**,**c**) obtained by PUMA (clique potential exponent is 0.5). (**h**,**i**) Errors between (**d**,**e**) and PU results (**f**,**g**). (**j**,**k**) PU results of (**b**,**c**) obtained by DB TSPA. (**l**,**m**) Errors between (**d**,**e**) and PU results (**j**,**k**). (**n**,**o**) PU results of (**b**,**c**) obtained by DB TSPA-PUMA. (**p**,**q**) Errors between (**d**,**e**) and PU results (**n,o**).

The third experiment is carried out on a real TanDEM-X DB InSAR dataset with two interferograms (single-pass) of Weinan of Shaanxi province, China. Figure 6a is the Google Earth image of the study area (1000×1000 pixels). We can see that Figure 6a is the area whose topography is mountainous and rugged. Under this condition, the phase continuity assumption may not work well, which causes that the SB PU cannot obtain the correct PU solution. Figure 6b,c are the flattened and filtered input interferograms with short and long baseline lengths, respectively. The major interferometric parameters of Figure 6b,c are listed in Table 4. Figure 6c,d are the corresponding reference unwrapped phase of Figure 6b,c, which are generated by the Shuttle Radar Topography Mission (SRTM) digital elevation model (DEM). Figure 6f,g show the PU results of Figure 6b,c obtained by the PUMA method with potential exponent 0.5, and Figure 6h,i are the errors between Figure 6d,e and Figure 6f,g, respectively. Figure 6j,k are the PU results of Figure 6b,c obtained by TSPA, and Figure 6l,m are the errors between Figure 6d,e and Figure 6j,k, respectively. Figure 6n,o are the PU results of Figure 6b,c obtained by TSPA-PUMA, and the corresponding errors between Figure 6d,e and Figure 6n,o is shown in Figure 6p,q, respectively. The statistical information of Figure 6 is listed in Table 5. From Table 5, we can see that when the baseline length is short and the fringe pattern in the interferogram is simple, the PU performance of all three methods is similar to each other. However, for the long baseline interferogram, because the phase variation is rapid, which does not obey the phase continuity assumption, the PU performance of TSPA and TSPA-PUMA are much better than that of PUMA. Although the PU results of TSPA and TSPA-PUMA are mainly the same due to their same $L^{1}$-norm model, their performances in terms of running time are not comparable. In this experiment, while TSPA-PUMA only takes 65.81 s for short baseline and 231.72 s for long baseline, the classical TSPA method is ten and eight times slower for the short and long baseline, respectively. Therefore, it can be seen that the running time of TSPA-PUMA is practical.

### 4.4. Experiment 4

**Figure 7:**
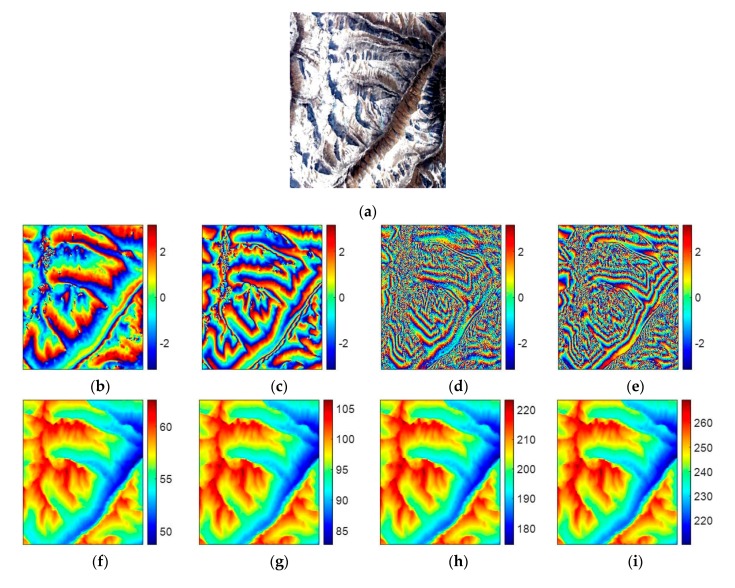

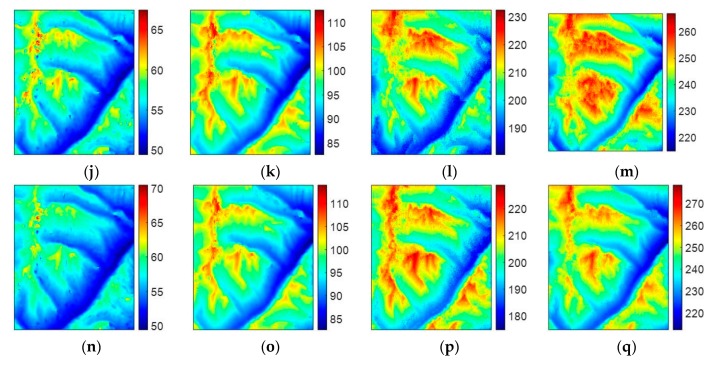
(**a**) Google Earth image of the study area. (**b**–**e**) ALOS PALSAR interferograms with different baseline lengths ((**b**) interferogram 1, (**c**) interferogram 2, (**d**) interferogram 3, and (**e**) interferogram 4). (**f**–**i**) The reference unwrapped phases of (**b**–**e**). (**j**–**m**) PU results of (**b**–**e**) obtained by PUMA (clique potential exponent is 0.5). (**n**–**q**) PU results of (**b**–**e**) obtained by MB TSPA-PUMA.

**Figure 8:**
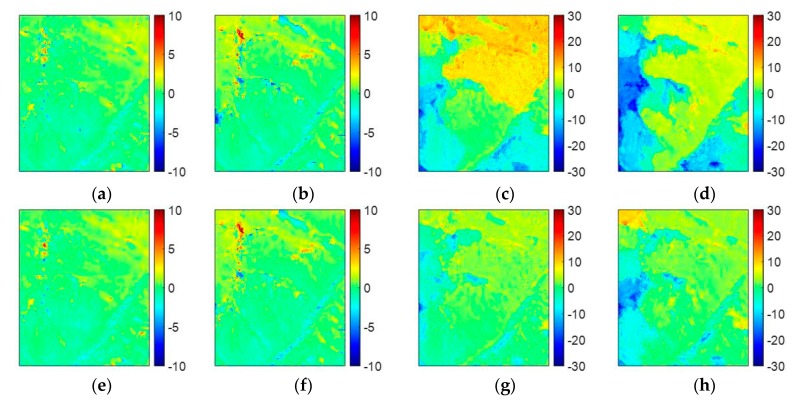
(**a**–**d**) PUMA errors of Figure 7j–m. (**e**–**h**) MB TSPA-PUMA errors of Figure 7n–q.

In the fourth experiment, we will examine the effectiveness of TSPA-PUMA in the real MB dataset of ALOS PALSAR with four interferograms. Figure 7a shows the Google Earth image of the study area in this experiment, which comes from the Himalayan mountain area. Figure 7b–e are four interferograms with different baseline lengths (601×501 pixels). From Figure 7d,e, we can observe that the coherence values of the two interferograms with long baseline are relatively low, because ALOS PALSAR acts as a repeat-pass radar interferometer with the inherent accuracy limitations imposed by temporal decorrelation and atmospheric disturbances. The major interferometric parameters of the ALOS PALSAR dataset are tabulated in Table 6. Figure 7f–i are the corresponding reference unwrapped phase of Figure 7b–e, which are obtained from the PALSAR DEM. Figure 7j–m are the PU results of Figure 7b–e generated by the PUMA method with potential exponent 0.5, and Figure 7n–q are those generated by TSPA-PUMA. Figure 8 show the corresponding errors between the PU results of PUMA, TSPA-PUMA and reference unwrapped phase. The statistical information of Figure 8 is listed in Table 7. From Table 7, it can be seen that, for the short baseline interferogram (Figure 7b,c), the PU results of the two methods are similar to each other. However, for the long baseline interferogram (Figure 7d,e), the PU performance of TSPA-PUMA is much better than that of PUMA, and reason is that TSPA-PUMA can break through the limitation of the phase continuity assumption. Also, TSPA-PUMA can eliminate the effects of low coherence through using the MB InSAR dataset with different baseline lengths.

### 4.5. Experiment 5

**Figure 9:**
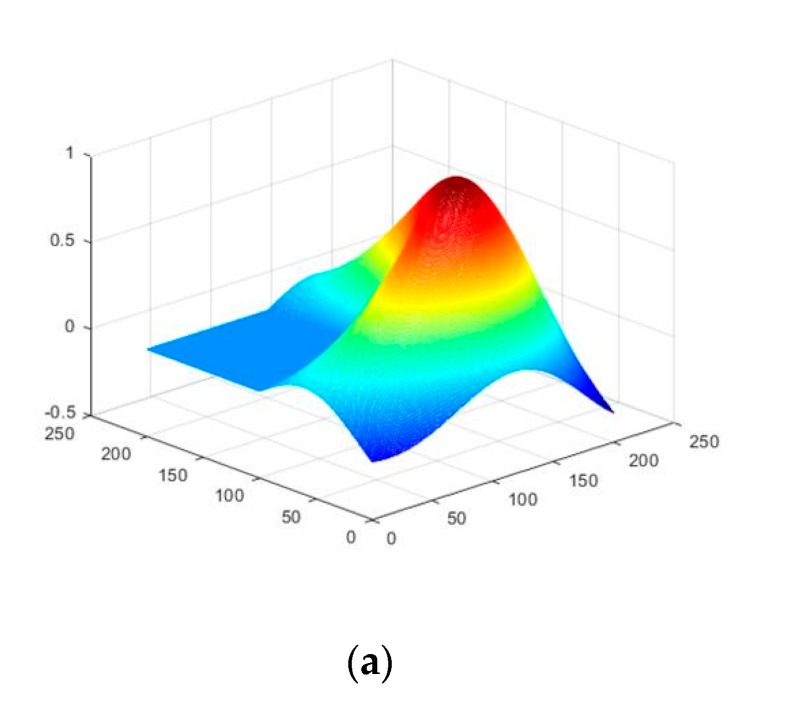

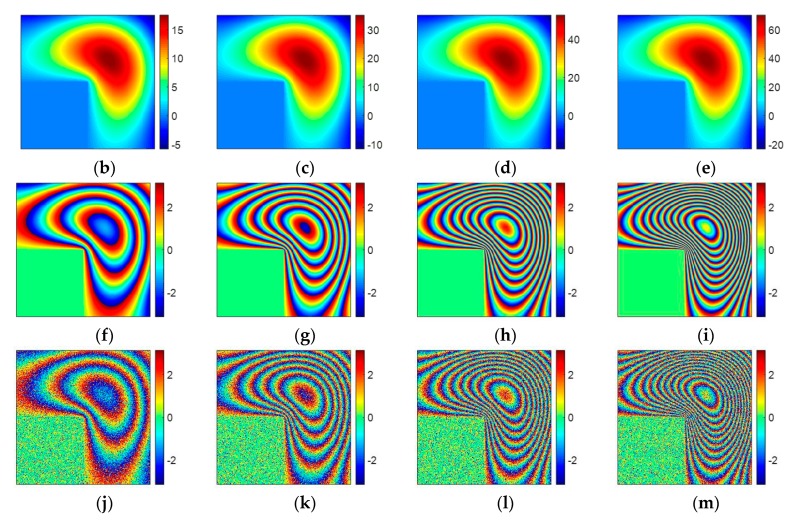
(**a**) The examples of simulated terrain obtained by the function membrane in 3D space. (**b**–**e**) reference unwrapped phase with four different ds ((**b**)$\;d_{1}=17.5,\;\left(c\right)\;d_{2}=35.0,\left(d\right)\;d_{3}=52.5,\left(e\right)\;d_{4}=70.0$, and unit is radian). (**f**–**i**) The simulated noise-free wrapped phases of (**b**–**e**). (**j**–**m**) The simulated wrapped phases of (**b**–**e**) with mean coherence coefficient 0.75.

**Figure 10:**
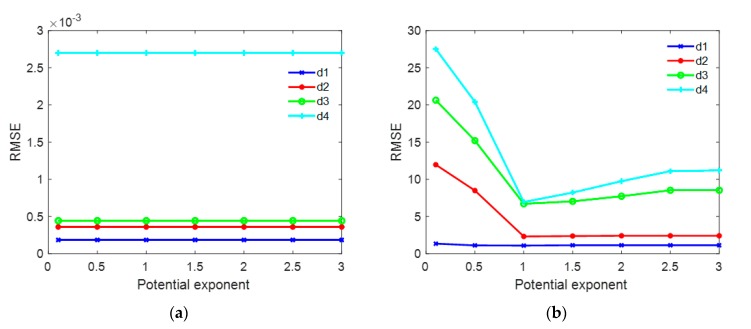
(**a**) RMSE curves of TSPA-PUMA of Figure 9f–i with different potential exponents between 0.1 and 3 with an increment of 0.5. (**b**) RMSE curves of TSPA-PUMA of Figure 9j–m with different potential exponents between 0.1 and 3 with an increment of 0.5.

In the last experiment, we explored the effect of the potential exponent p in stage 2 of TSPA-PUMA method on the simulated MB InSAR dataset. This experiment examined the PU performance of TSPA-PUMA with different potential exponent p ranging from 0.1 to 3 with an increment of 0.5. A simulated terrain generated by the MATLAB’s membrane function was used to test the relationship between the potential exponent p and the terrain change. Figure 9a shows the simulated terrain employed in this experiment (201×201 pixels). According to the simulated terrain, we generated the reference unwrapped phases using *d* × membrane, where *d* is the parameter that determines the height of the terrain. The larger the value of *d* is, the higher the terrain is, and thus the terrain changes more violently. We considered four MB simulated reference unwrapped phases with different *d*s (i.e., $d_{1}=17.5,\;d_{2}=35.0,\;d_{3}=52.5,\;d_{4}=70.0$, and unit is radian). Figure 9b–e show four reference unwrapped phases with different ds, respectively. From Figure 9b–e, we observe that, while the value of d is getting larger, and the pattern of the fringe becomes denser, which results in the failure of the phase continuity assumption. We generated two groups of simulated interferograms of Figure 9b–e. In one group, we simulated four noise-free wrapped phase images, as shown in Figure 9f–i, respectively. Under the noise-free condition, the fringe change of Figure 9f–i is only related to the topography changes. In this case, we can test the dependence of the potential exponent p on the steepness of the terrain. In another group, we simulated four noisy wrapped phase images, in which the phase noise was added with using 0.75 mean correlation coefficient [35], as illustrated in Figure 9j–m, respectively. From Figure 9j–m, it can be seen that the pattern of the fringe is destroyed more after the noise is added. Under this condition, we can examine the effect of the potential exponent p in case of high-phase noise. We compare the unwrapped phases obtained by TSPA-PUMA using different potential exponent p with the reference unwrapped phases of Figure 9b–e and obtain the RMSE of each PU result. Figure 10a shows the RMSE curves of Figure 9f–i with different ds, and Figure 10b is the RMSE curves of Figure 9j–m with different ds. From the trends of the curves shown in Figure 10a, we can see that the RMSE curves of the PU results with four different ds are low (below $3\times 10^{-3}$) and identical throughout the whole potential exponent scale, meaning that the potential exponent p is not sensitive to the terrain change. The reason is that, owning to stage 1 of TSPA-PUMA without obeying the phase continuity assumption, no matter what kind of the potential exponent value is chosen in stage 2, it is possible for TSPA-PUMA to perform correctly. From the trends of the curves shown in Figure 10b, we can observe that the PU results with four different ds generate the lowest RMSE when the potential exponent equals to 1 (p=1), while when the potential exponent more than 1 or less than 1 (p>1 or p<1), the PU results both have higher RMSE with the different values ds. This is because that when p>1, TSPA-PUMA introduces the incorrect phase gradients from the low-quality regions into the high-quality regions easily, and when the potential exponent is less than 1 (p<1), the clique potential of TSPA-PUMA grows much more slowly than that when potential exponent equals to 1 (p=1), which allows strong phase noise not be too much penalized. It implies that the potential exponent p which equals to 1 is the optimal parameter for TSPA-PUMA both in the rugged and low-quality regions.

## 5. Conclusions

In this paper, we extend the classical PUMA algorithm for MB InSAR using the TSPA approach referred to as TSPA-PUMA, consisting of a two-stage programming procedure. In stage 1 of TSPA-PUMA, the phase gradients are estimated based on CRT, which does not follow the phase continuity assumption. In stage 2, an MRF model of PUMA is designed for modeling local contextual dependence based on the phase gradients obtained by stage 1. Subsequently, the energy of MRF model is minimized by performing a sequence of binary optimizations solved by graph cuts techniques. Results of the simulated and real InSAR data experiments demonstrate that the TSPA-PUMA method can significantly improve the accuracy of the original PUMA method in the area where topography varies drastically due to its ability to overcome the limitation of the phase continuity assumption, and is an efficient MB PU method compared to the original TSPA method. In addition, the noise robustness of TSPA-PUMA can also be improved through adding more interferograms with different baseline lengths.

## Figures and Tables

**Table 1:** Major parameters of simulated InSAR system and Interferograms.

**Orbit Altitude**	**Incidence Angle**	**Wavelength**
6885 km	46°	0.031 m
**Interferogram**	**Baseline Length**	
Figure 1c	150 m	
Figure 1d	330 m	

**Table 2:** Statistical information of PU performance in Figure 1g,h,k,l, and Figure 4g,h.

U Method	Short Baseline		Long Baseline	
	Figure	RMSE	Figure	RMSE
PUMA with potential exponent 1	Figure 1g	0.9505	Figure 1k	3.6438
PUMA with potential exponent 0.5	Figure 1h	0.9505	Figure 1l	3.2356
TSPA-PUMA	Figure 4g	0.9164	Figure 4h	0.9164

**Table 3:** The relationship between the estimation RMSE of TSPA-PUMA and the number of interferograms.

ID	Number of Interferograms	Baseline Length (m)								RMSE
1	2	150	330							7.6592
2	3	70	150	330						6.9732
3	4	70	150	330	471					6.7486
4	5	70	150	330	471	550				6.6240
5	6	70	150	330	471	550	631			4.6023
6	7	70	150	330	471	550	631	753		4.3318
7	8	70	150	330	471	550	631	753	831	3.4297

**Table 4:** Major interferometric parameters of real DB dataset of TanDEM-X.

Orbit Altitude	Incidence Angle	Wavelength	Latitude	longitude
514.8 km	36.6°	0.032 m	35.82°	109.28°
**Interferogram**	Figure 6b		Figure 6c	
**Date of Master Channel**	2 April 2014		21 October 2012	
**Date of Slave Channel**	2 April 2014		21 October 2012	
**Baseline Length**	130.62 m		370.45 m	
**Resolution**	Range (Vertical)	5.46 m	Azimuth (Horizontal)	8.15 m
**Image Size**	Range	1000 pixels	Azimuth	1000 pixels

**Table 5:** Statistical information of PU performance in Figure 6h,i,l,m,p,q.

PU Method	Short Baseline			Long Baseline		
	Figure	RMSE	Time (s)	Figure	RMSE	Time (s)
PUMA with potential exponent 0.5	Figure 6h	0.6871	116.18	Figure 6i	10.0892	303.83
TSPA	Figure 6l	0.6114	665.92	Figure 6m	1.91	1941.04
TSPA-PUMA	Figure 6p	0.69	65.81	Figure 6q	1.7616	231.72

**Table 6:** Major interferometric parameters of real MB dataset of ALOS PALSAR.

Orbit Altitude	Incidence Angle	Wavelength	Latitude	longitude
698.51 km	38.75°	0.236m	30.91°	94.23°
**Interferogram**	Figure 7b	Figure 7c	Figure 7d	Figure 7e
**Date of Master Channel**	18 August 2007	18 August 2007	18 August 2007	18 August 2007
**Date of Slave Channel**	3 October 2007	3 July 2007	3 January 2008	8 October 2009
**Baseline Length**	113.36 m	193.15 m	406.00 m	440.68 m
**Resolution**	Range (Vertical)	9.37 m	Azimuth (Horizontal)	19.00 m
**Image Size**	Range	601 pixels	Azimuth	501 pixels

**Table 7:** Statistical information of PU performance in Figure 8a–h.

PU Method	Interferogram 1		Interferogram 2		Interferogram 3		Interferogram 4	
	Figure	RMSE	Figure	RMSE	Figure	RMSE	Figure	RMSE
PUMA with potential exponent 0.5	Figure 8a	0.8719	Figure 8b	1.1576	Figure 8c	7.4004	Figure 8d	7.4951
TSPA-PUMA	Figure 8e	0.8577	Figure 8f	1.0860	Figure 8g	3.4688	Figure 8h	4.6751
